# Risk factors of undiagnosed and uncontrolled hypertension in primary care patients with hypertension: a cross-sectional study

**DOI:** 10.1186/s12875-024-02511-4

**Published:** 2024-08-20

**Authors:** Emmanuel Adediran, Robert Owens, Elena Gardner, Andrew Curtin, John Stuligross, Danielle Forbes, Jing Wang, Dominik Ose

**Affiliations:** 1 Department of Family and Preventive Medicine, University of Utah, Salt Lake City, UT USA; 2 Faculty of Health and Healthcare Sciences, Westsächsische Hochschule Zwickau, Kornmarkt 1, 08056 Zwickau, 08012 Zwickau, Saxony, Germany; 3 Utah Department of Health and Human Services, Salt Lake City, UT USA; 4 Intermountain Healthcare, Salt Lake City, UT USA

**Keywords:** Undiagnosed hypertension, Uncontrolled hypertension, Primary care, Black/African americans, Native Hawaiian/Pacific Islander

## Abstract

**Background:**

Hypertension is a common heart condition in the United States (US) and severely impacts racial and ethnic minority populations. While the understanding of hypertension has grown considerably, there remain gaps in US healthcare research. Specifically, there is a lack of focus on undiagnosed and uncontrolled hypertension in primary care settings.

**Aim:**

The present study investigates factors associated with undiagnosed and uncontrolled hypertension in primary care patients with hypertension. The study also examines whether Black/African Americans are at higher odds of undiagnosed and uncontrolled hypertension compared to White patients.

**Methods:**

A cross-sectional study was conducted using electronic health records (EHR) data from the University of Utah primary care health system. The study included for analysis 24,915 patients with hypertension who had a primary care visit from January 2020 to December 2020. Multivariate logistic regression assessed the odds of undiagnosed and uncontrolled hypertension.

**Results:**

Among 24,915 patients with hypertension, 28.6% (*n* = 7,124) were undiagnosed and 37.4% (*n* = 9,319) were uncontrolled. Factors associated with higher odds of undiagnosed hypertension included age 18–44 (2.05 [1.90–2.21]), Hispanic/Latino ethnicity (1.13 [1.03–1.23]),  Medicaid (1.43 [1.29-1.58]) or self-pay  (1.32 [1.13-1.53]) insurance, CCI 1-2 (1.79 [1.67-1.92]), and LDL-c ≥ 190 mg/dl (3.05 [1.41–6.59]). For uncontrolled hypertension, risk factors included age 65+ (1.11 [1.08–1.34]), male (1.24 [1.17–1.31]), Native-Hawaiian/Pacific Islander (1.32 [1.05-1.62])  or Black/African American race (1.24 [1.11-1.57]) , and self-pay insurance (1.11 [1.03-1.22]).

**Conclusion:**

The results of this study suggest that undiagnosed and uncontrolled hypertension is prevalent in primary care. Critical risk factors for undiagnosed hypertension include younger age, Hispanic/Latino ethnicity, very high LDL-c, low comorbidity scores, and self-pay or medicaid insurance. For uncontrolled hypertension, geriatric populations, males, Native Hawaiian/Pacific Islanders, and Black/African Americans, continue to experience greater burdens than their counterparts. Substantial efforts are needed to strengthen hypertension diagnosis and to develop tailored hypertension management programs in primary care, focusing on these populations.

**Supplementary Information:**

The online version contains supplementary material available at 10.1186/s12875-024-02511-4.

## Introduction

Hypertension is a common heart health condition affecting 1.2 billion adults worldwide [1] and more than 119 million adults in the United States (US) [2]. Hypertension is also a contributing risk factor for stroke, dementia, and all-cause mortality in the US [3–5].

Importantly, hypertension varies by demographics. A majority of US adults with diagnosed hypertension are male, older age, and identify as Black/African American or Asian/Asian American [6–8]. Black/African Americans, in particular, experience an even greater burden of hypertension [8], often diagnosed at a younger age.

While the understanding of hypertension has grown considerably, there remain gaps in US healthcare research. Specifically, there is limited focus on undiagnosed and uncontrolled hypertension in primary care [9]. Additionally, hypertension prevalence in US primary care settings has not been widely established or confirmed. A 2017 study indicated a prevalence of 25% for uncontrolled hypertension and 46.9% for overall hypertension (including controlled) [10]. However, the results are limited by a potential measurement bias, suggesting further research is needed.

Even less is known about undiagnosed hypertension. It is estimated 11 million adults in the US suffer from hypertension without a clinical diagnosis [11, 12]. Prior studies have also reported hypertension underdiagnosis in the health records and identified coding errors, gaps in knowledge of guidelines, and variations in healthcare delivery, as contributing factors [12, 13].

The role of health systems in addressing hypertension disparities remains a topic of ongoing debate. Primary care, for example, offers a comprehensive, patient-centered, and community-based care model, which serves as a bridge to affordability and increased outreach to promote early disease detection and prevention [9, 14], and may be a promising long-term solution.

However, healthcare and research institutions have yet to leverage primary care fully to improve hypertension. So far, discussions on undiagnosed hypertension have remained scarce [15]. The lack of knowledge, in particular regarding undiagnosed hypertension equates to missed opportunities for early identification of at-risk populations and for developing interventions for those populations.

To fill this gap, this study investigates undiagnosed and uncontrolled hypertension risk factors in primary care patients with hypertension. The study also examines whether Black/African Americans are at higher odds of undiagnosed and uncontrolled hypertension compared to White patients.

## Methods

### Study design and setting

The present study was reported following the Strengthening the Reporting of Observational Epidemiology (STROBE) methodology for cross-sectional studies [16]. The study utilized clinical data from the University of Utah (UU) primary care system in Salt Lake City, Utah. Overall, the UU health system comprises 5 hospitals and 12 primary care clinics.

### Study population and selection

This cross-sectional study retrieved clinical data (January 1, 2020 – December 31, 2020) from all 12 primary care clinics. All clinics operate a shared electronic health records (EHR) system. Patient’s demographics data were extracted, including age, sex, race and ethnicity, Body Mass Index (BMI), insurance provider, Charlson Comorbidity Index (CCI), controlled/uncontrolled hypertension status, and diagnosed/undiagnosed hypertension status, diabetes and pre-diabetes status, blood glucose levels, and blood lipid profiles (e.g., dyslipidemia, low-density lipoprotein cholesterol, and hypercholesterolemia).

### Inclusion and exclusion criteria

**Fig. 1:**
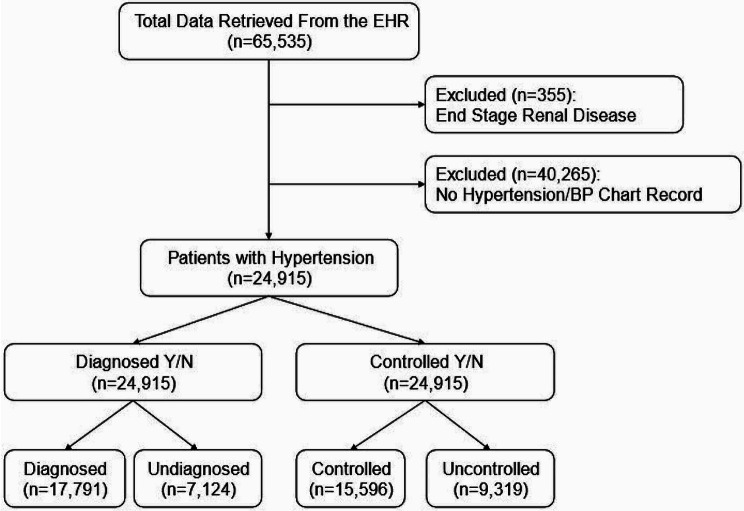
Flow chart of included data

A total of 65,535 patient’s records (18+) were retrieved from the EHR. Next, 355 patients diagnosed with end-stage renal disease (ESRD) were excluded, leaving 65,180 unique records. Patients with ESRD were excluded based on research suggesting renal disease as a primary case of high blood pressure in patients with ESRD and hypertension comorbidities [17]. Then, 40,265 patients with no hypertension/blood pressure (BP) chart record in the EHR were excluded, leaving 24,915 patients with hypertension (diagnosed and undiagnosed; controlled and uncontrolled) for analysis (Fig. 1).

### Sample size calculation

In the present study, the expected hypertension prevalence was 30%, calculated from the total patient population (24,915/65,535). The significance level is 5% (i.e., a 95% confidence interval) and power of 80%. Power analysis showed a sample size of 384 would be needed to observe a hypertension prevalence of 30% with a significance level of 5% and power of 80%.

### Study measures

#### Outcomes

Undiagnosed and uncontrolled hypertension were two binary outcome variables. Undiagnosed hypertension is defined as patients (18+) who have hypertension based on vitals (lab values) or medication prescription but no ICD (international classification of diseases) code (see codebook) [18].

Uncontrolled hypertension includes patients 18 years and older with hypertension diagnosis (based on ICD, vitals, prescribed medication) and whose latest BP reading in 2020 is systolic ≥ 140 mmHg and diastolic ≥ 90 mmHg. For hypertension diagnosis, the UofU health utilizes an in-house guideline which looks for most recent office BP ≥ 140/90 mm Hg, and checks if most recent automated office blood pressure reading (AOBP) ≥ 135/85 mm Hg, and if most recent 24-hr average BP ≥ 130/80 mm Hg, and if most recent average home BP reading ≥ 135/85 mm Hg.

#### Independent variables

The independent variables consisted of sex (Male; Female), age (18–44; 45–64; 65+), race (White; Asian; Black/African American; Native Hawaiian/Pacific Islander; American Indian/Alaska Native; Other/Unknown), Ethnicity (Non-Hispanic/Latino; Hispanic/Latino, Unknown), BMI (Underweight < 18.5 Kg/m^2^; Healthy weight 18.5-24.99 Kg/m^2^; Overweight 25.0-29.99 Kg/m^2^; Obesity Class 1 30.0-34.99 Kg/m^2^; Obesity Class 2 35.0–39.00 Kg/m^2^; and Obesity Class 3 40.0 + Kg/m^2^) [19], insurance provider (UT commercial; UT Medicare; UT Medicaid; Self-pay; Other), CCI (None (0); Mild (1–2); Moderate (3–4); Severe (5–21); and unknown) [20], current statin use (Yes/No), diabetes status (based on ICD (Yes/No) and pre-diabetes (Yes/No), blood glucose level status (HbA1c control based on ICD (Yes/No)). Other medical conditions included: dyslipidemia (Yes/No), hypercholesterolemia (Yes/No), and low-density lipoprotein cholesterol (very high LDL-c ≥ 190 mg/dl (Yes/No); LDL-c between 70 and 189 mg/dl (Yes/No)).

### Statistical analysis

All statistical analyses were performed using RStudio build 481 (Posit Software, PBC). Study variables were first reviewed for completeness, by identifying missing values and incorrect responses. Frequencies (n) and relative frequencies (%) were calculated for categorical variables and means and standard deviation (SD) for continuous variables. Group differences for sample characteristics were also tested using chi-squared tests. The frequency distributions by undiagnosed and uncontrolled hypertension are presented in Table 1.

**Table 1:** Characteristics of primary care patients by undiagnosed and uncontrolled hypertension

	Hypertension	Undiagnosed Hypertension			Uncontrolled Hypertension		
Characteristics	**Overall** **N (%)**	**Undiagnosed** **N (%)**	**Diagnosed** **N (%)**	**P value** ^**a, c**^	**Uncontrolled N (%)**	**Controlled** **N (%)**	**P value** ^**a, c**^
Included in Analysis	24,915	7,124 (28.6)	17,791 (71.4)		9,319 (37.4)	15,596 (62.6)	
Age (years) mean (SD)^c^	57.8 (16.3)	49.3 (16.3)	61.1 (14.9)	< 0.001	57.9 (16.2)	57.6 (16.3)	< 0.001
18–44 years old	5,643 (22.7)	2,911 (51.6)	2,732 (48.4)	< 0.001	2,096 (37.1)	3,547 (62.9)	< 0.001
45–64 years old	9,770 (39.2)	2,764 (28.3)	7,006 (71.7)		3,690 (37.8)	6,080 (62.2)	
65 + years old	9,502 (38.1)	1,449 (15.2)	8,053 (84.8)		3,533 (37.2)	5,969 (62.8)	
Sex^b^				< 0.001			< 0.001
Female	12,867 (51.6)	3,664 (28.5)	9,203 (71.5)		4,504 (35.0)	8,363 (65.0)	
Male	12,045 (48.3)	3,458 (28.7)	8,587 (71.3)		4,814 (40.0)	7,231 (60.0)	
Race				< 0.001			< 0.001
White	19,671 (78.9)	5,540 (28.2)	14,131 (71.8)		7,220 (36.7)	12,451 (63.3)	
Asian	746 (2.9)	200 (26.8)	546 (73.2)		299 (40.1)	447 (59.9)	
Black/African American	668 (2.6)	186 (27.8)	482 (72.2)		284 (42.5)	384 (57.5)	
Native Hawaiian/Other Pacific	421 (1.6)	132 (31.4)	289 (68.6)		189 (44.9)	232 (55.1)	
American Indian/Alaska Native	180 (0.7)	53 (29.4)	127 (70.6)		59 (32.8)	121 (67.2)	
Other/Unknown	3,229 (12.9)	1,013 (31.4)	2,216 (68.6)		1,268 (39.3)	1,961 (60.7)	
Ethnicity				< 0.001			< 0.001
Non-Hispanic/Latino	21,302 (85.4)	5,945 (27.9)	15,357 (72.1)		7,939 (37.3)	13,363 (62.7)	
Hispanic/Latino	3,092 (12.4)	1,007 (32.6)	2,085 (67.4)		1,174 (38.0)	1,918 (62.0)	
Unknown	521 (2.1)	172 (33.0)	349 (67.0)		206 (39.5)	315 (60.5)	
BMI (kg/m^2^) mean (SD)^c^	31.3 (7.8)	29.1 (7.4)	31.6 (7.7)	< 0.001	31.4 (7.8)	29.4 (7.3)	0.338
Underweight (< 18.5)	247 (0.9)	74 (30.0)	173 (70.0)	< 0.001	114 (46.2)	133 (53.8)	< 0.001
Healthy Weight (18.5–24.99)	3,324 (13.3)	1,348 (40.6)	1,976 (59.4)		1,056 (31.8)	2,268 (68.2)	
Overweight (25.0–29.99)	6,340 (25.4)	1,968 (31.0)	4,372 (69.0)		295 (4.7)	6,045 (95.3)	
Obesity Class 1 (30.0–34.99)	5,123 (20.6)	1,437 (28.0)	3,686 (72.0)		1,711 (33.4)	3,412 (66.6)	
Obesity Class 2 (35.0–39.99)	2,851 (11.4)	828 (29.0)	2,023 (71.0)		929 (32.6)	1,922 (67.4)	
Obesity Class 3 (40.0+)	2,691 (10.8)	740 (27.5)	1,951 (72.5)		875 (32.5)	1,816 (67.5)	
Unknown	4,339 (17.4)	729 (16.8)	3,610 (83.2)		4,339 (100)	0	
Insurance				< 0.001			< 0.001
UT Commercial	12,126 (48.6)	4,226 (34.9)	7,900 (65.1)		4,673 (38.5)	7,453 (61.5)	
UT Medicare	9,454 (37.9)	1,493 (15.8)	7,961 (84.2)		3,368 (35.6)	6,086 (64.4)	
UT Medicaid	2,234 (8.9)	943 (42.2)	1,291 (57.8)		773 (34.6)	1,461 (65.4)	
Self-pay	908 (3.6)	380 (41.9)	528 (58.1)		438 (84.2)	470 (51.8)	
Other	193 (0.8)	82 (42.5)	111 (57.5)		67 (34.7)	126 (65.3)	
CCI							
None (0)	7,645 (30.6)	3,439 (45.0)	4,206 (55.0)	< 0.001	3,304 (43.2)	4,341 (56.8)	< 0.001
Mild (1–2)	8,784 (35.2)	2,561 (29.2)	6,223 (70.8)		3,239 (36.9)	5,545 (63.1)	
Moderate (3–4)	4,162 (16.7)	686 (16.5)	3,476 (83.5)		1,397 (33.6)	2,765 (66.4)	
Severe (5–21)	4293 (17.2)	429 (10.0)	3,864 (90.0)		1,348 (31.4)	2,945 (68.6)	
Unknown	31 (0.1)	10 (32.3)	21 (67.7)		10 (32.3)	21 (67.7)	
Current Statin Use	57.8 (16.3)	49.3 (16.3)	61.1 (14.9)	< 0.001	57.9 (16.2)	57.6 (16.3)	< 0.001
Yes	9,881 (39.6)	1,717 (17.4)	8,164 (82.6)	< 0.001	3,385 (34.3)	6,496 (65.7)	< 0.001
No	15,034 (60.4)	5,407 (36.0)	9,627 (64.0)		5,934 (39.5)	9,100 (60.5)	
Diabetes							
Diabetes ICD (Yes)	6,489 (26.0)	655 (10.1)	5,834 (89.9)	< 0.001	2,146 (33.1)	4,343 (66.9)	< 0.001
No	18,426 (74.0)	6,469 (35.1)	11,957 (64.9)		7,173 (38.9)	11,253 (61.1)	
Pre-Diabetes (Yes)	2,767 (11.1)	740 (26.7)	2,027 (73.3)	0.022	936 (33.8)	1,831 (66.2)	< 0.001
No	22,148 (88.9)	6,384 (28.8)	15,764 (71.2)		8,383 (37.8)	13,765 (62.2)	
Blood Glucose Levels							
Controlled HbA1c ICD (Yes)	4,360 (17.4)	415 (9.5)	3,945 (90.5)	< 0.001	1,311 (30.1)	3,049 (69.9)	< 0.001
No	20,555 (82.6)	6,709 (32.6)	13,846 (67.4)		8,008 (39.0)	12,547 (61.0)	
Other Metabolic Conditions							
Dyslipidemia (Yes)	6,648 (26.6)	508 (7.6)	6,140 (92.4)	< 0.001	2,298 (34.6)	4,350 (65.4)	< 0.001
No	18,267 (73.4)	6,616 (36.2)	11,651 (63.8)		7,021 (38.4)	11,246 (61.6)	
LDL-c-HIGH (> 190 mg/dl) (Yes)	34 (0.1)	18 (5.3)	16 (94.7)	< 0.001	12 (35.3)	22 (64.7)	0.799
No	24,881 (99.0)	7,106 (28.6)	17,775 (71.4)		9,307 (37.4)	15,574 (62.6)	
LDL-c-70_189 mg/dl (Yes)	1,194 (4.8)	267 (22.4)	927 (77.6)	< 0.001	451 (37.8)	743 (62.2)	0.787
No	23,721 (95.2)	6,857 (28.9)	16,864 (71.1)		8,868 (37.4)	14,853 (62.6)	
Hypercholesterolemia (Yes)	6,401 (25.6)	588 (9.2)	5,813 (90.8)	< 0.001	2,219 (34.7)	4,182 (65.3)	< 0.001
No	18,514 (74.4)	6,536 (35.3)	11,978 (64.7)		7,100 (38.3)	11,414 (61.7)	

Abbreviations: SD: Standard Deviation; BMI: Body mass index; CCI: Charlson comorbidity index; ICD: International classification of diseases (ICD); HbA1c: Hemoglobin A1c tests; LDL-c: Low-density lipoprotein; ^a^ Chi-Square test for independence, ^b^ There were an additional 3 cases of reported sex “other,” ^c^ Results of Two-sample T-test

**Fig. 2:**
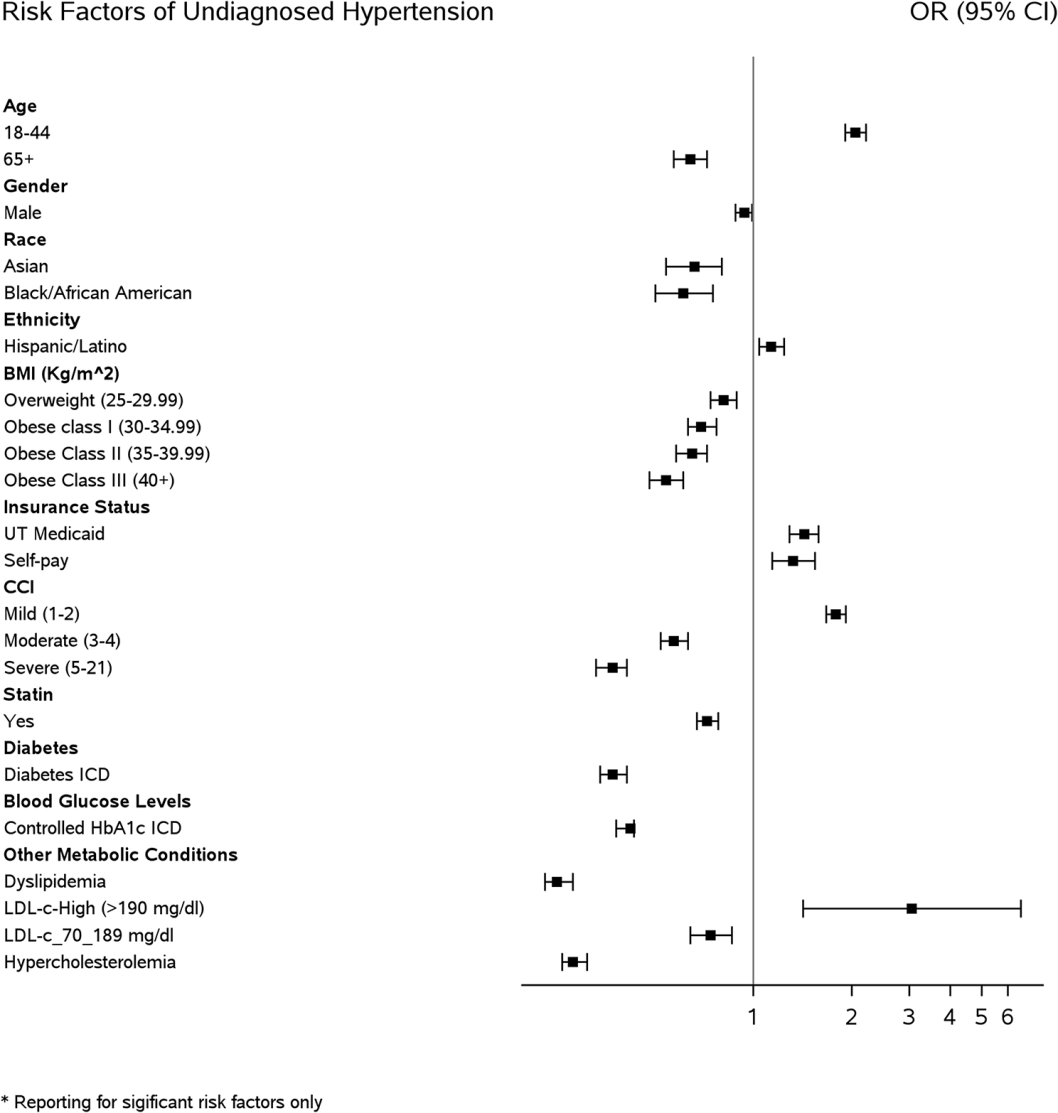
Forest plot of significant risk factors of undiagnosed hypertension

**Fig. 3:**
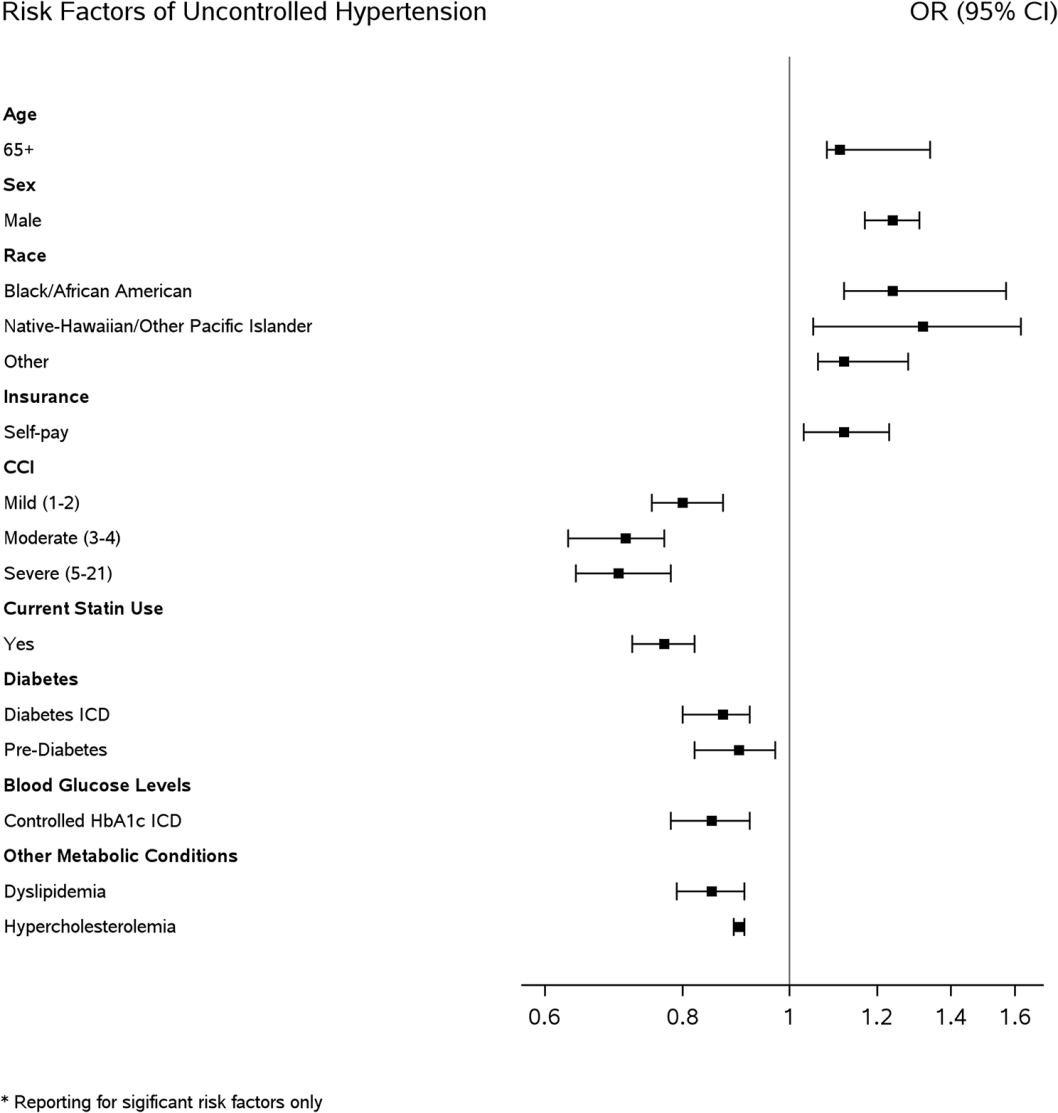
Forest plot of significant risk factors of undiagnosed hypertension: Black/African Americans vs. White

**Fig. 4:**
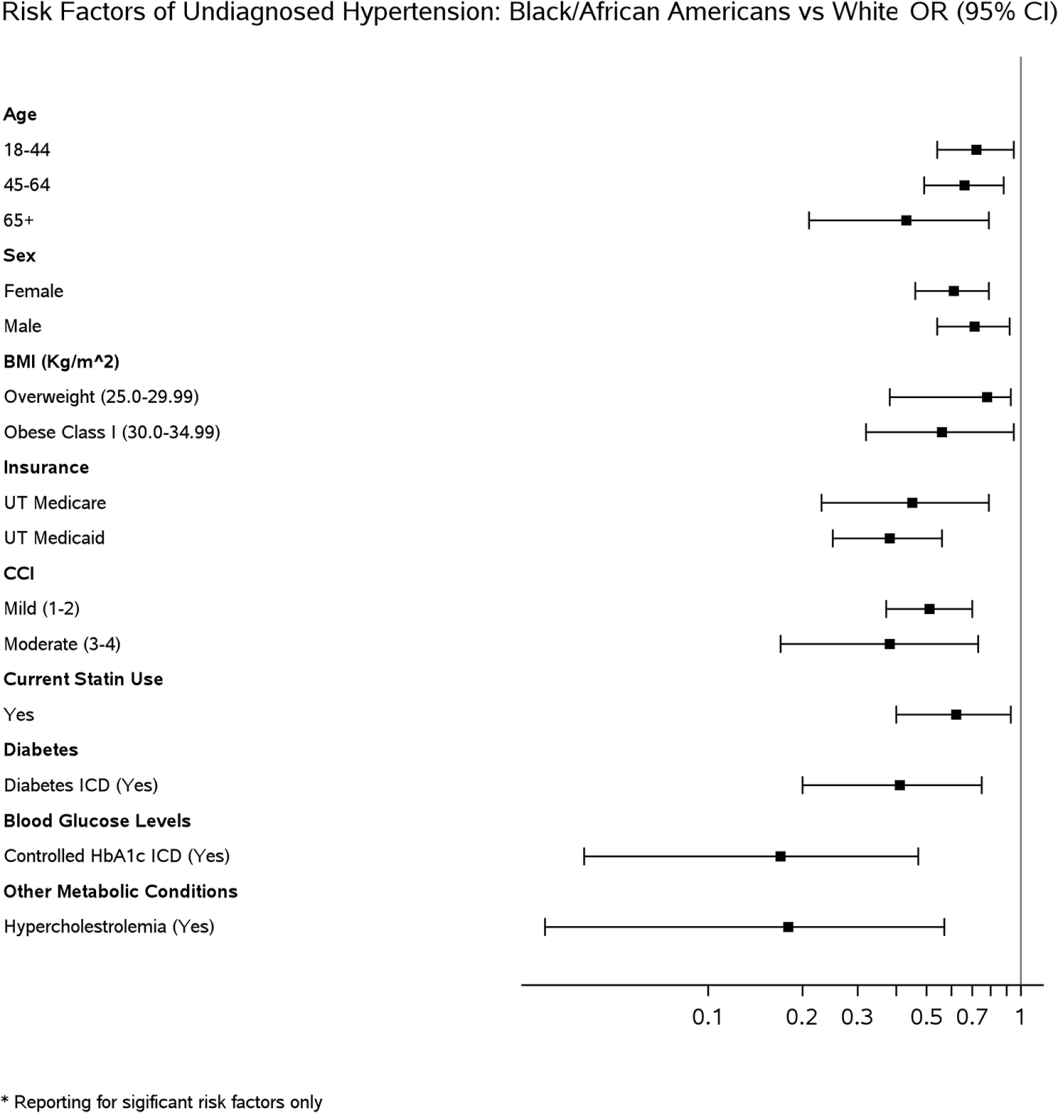
Forest plot of significant risk factors of uncontrolled hypertension

**Fig. 5:**
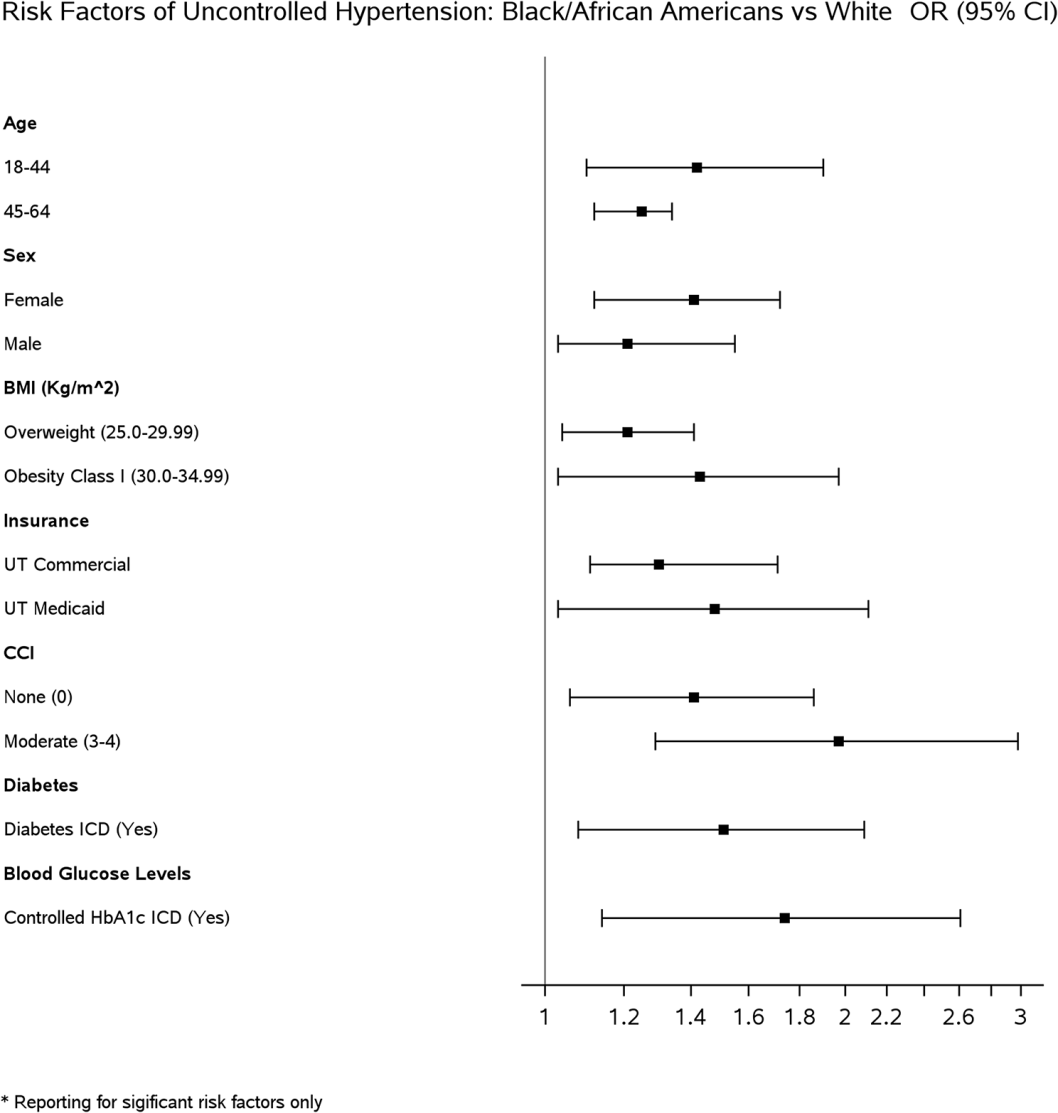
Forest plot of significant risk factors of uncontrolled hypertension: Black/African Americans vs. White

Multivariable logistic regression was used to evaluate the association between the independent variables and the odds of undiagnosed and uncontrolled hypertension while adjusting for race, ethnicity, sex, age, health insurance status, valid range BMI, and CCI. Adjusted odds ratios (aOR) were reported with the corresponding 95% confidence intervals (C.I.) and p-values to check the statistical significance (i.e., *P* < .05) (Tables 2, 3, and 4). Forest plots showing the aOR are provided in Figs. 2, 3 , 4 and 5.

**Table 2:** Risk factors of undiagnosed and uncontrolled hypertension in primary care

	Undiagnosed Hypertension			Uncontrolled Hypertension		
Risk Factors	**aOR**	**95% CI**	***P*** **values**	**aOR**	**95% CI**	***P*** **values**
Age						
45–64 years old	Reference					
18–44 years old	**2.053**	**1.905–2.212**	**< 0.001**	**0.960**	**0.903-1.028**	**0.258**
65 + years old	**0.640**	**0.573–0.715**	**< 0.001**	**1.112**	**1.081–1.342**	**< 0.001**
Sex						
Female	Reference					
Male	**0.939**	**0.882–0.999**	**0.048**	**1.242**	**1.174–1.314**	**< 0.001**
Race						
White	Reference					
Asian	**0.656**	**0.538–0.800**	**< 0.001**	1.122	0.952-1.319	0.152
Black/African American	**0.612**	**0.501–0.746**	**< 0.001**	**1.243**	**1.118-1.573**	**< 0.001**
Native Hawaiian/Other Pacific Islander	1.170	0.910–1.491	0.218	**1.321**	**1.053-1.621**	**< 0.001**
American Indian/Alaska Native	0.985	0.678–1.413	0.935	0.912	0.649-1.269	0.531
Other	0.985	0.858–1.130	0.826	**1.122**	**1.062-1.280**	**< 0.001**
Ethnicity						
Non-Hispanic/Latino	Reference					
Hispanic/Latino	**1.134**	**1.036–1.239**	**0.006**	1.072	0.952-1.219	0.241
BMI kg/m^2^						
Healthy weight (18.5-24.99)	Reference					
Underweight (< 18.5)	1.388	0.997–1.921	0.050	1.009	0.717-1.416	0.432
Overweight (25.0–29.99)	**0.814**	**0.740–0.895**	**< 0.001**	1.022	0.897-1.070	0.592
Obesity Class 1 (30.0–34.99)	**0.755**	**0.718-0.795**	**< 0.001**	1.045	0.984-1.105	0.132
Obesity Class 2 (35.0–39.99)	**0.763**	**0.686-0.850**	**< 0.001**	1.035	0.963-1.110	0.266
Obesity Class 3 (40.0+)	**0.677**	**0.608-0.754**	**< 0.001**	1.078	0.999-1.161	0.061
Insurance						
UT Commercial	Reference					
UT Medicare	0.898	0.805–1.003	0.056	0.920	0.837–1.010	0.081
UT Medicaid	**1.431**	**1.293–1.583**	**< 0.001**	0.946	0.853–1.047	0.283
Self-Pay	**1.322**	**1.137–1.536**	**< 0.001**	1.117	1.033-1.227	0.004
CCI						
None (0)	Reference					
Mild (1–2)	**1.795**	**1.674–1.925**	**< 0.001**	**0.804**	**0.745-0.868**	**< 0.001**
Moderate (3–4)	**0.573**	**0.519–0.633**	**< 0.001**	**0.707**	**0.631-0.770**	**<0.001**
Severe (5–21)	**0.367**	**0.326–0.412**	**< 0.001**	**0.704**	**0.636-0.779**	**<0.001**
Current Statin Use						
Yes	**0.720**	**0.670–0.774**	**< 0.001**	**0.769**	**0.721-0.819**	**< 0.001**
Diabetes						
Diabetes ICD (Yes)	**0.371**	**0.336–0.409**	**< 0.001**	**0.868**	**0.802-0.923**	**< 0.001**
Pre-Diabetes (Yes)	0.966	0.876–1.064	0.485	**0.899**	**0.823-0.970**	**0.011**
Blood Glucose Levels						
Controlled HbA1c ICD (Yes)	**0.421**	**0.375–0.472**	**< 0.001**	**0.848**	**0.780-0.915**	**< 0.001**
Other Metabolic Conditions						
Dyslipidemia (Yes)	**0.250**	**0.225–0.276**	**< 0.001**	**0.850**	**0.794–0.910**	**< 0.001**
LDL-c-HIGH (> 190 mg/dl) (Yes)	**3.050**	**1.416–6.596**	**0.004**	0.994	0.688-1.595	0.923
LDL-c-70_189 mg/dl (Yes)	**0.742**	**0.637–0.863**	**< 0.001**	1.090	0.963-1.232	0.188
Hypercholesterolemia (Yes)	**0.283**	**0.257–0.311**	**< 0.001**	**0.901**	**0.894-0.907**	**< 0.001**

Abbreviations: aOR, adjusted odds ratio; SD: Standard Deviation; BMI: Body mass index; CCI: Charlson comorbidity index; ICD: International classification of diseases (ICD); HbA1c: Hemoglobin A1c tests; LDL-c: Low-density lipoprotein: Logistic regression adjusted odds ratio estimate with 95% confidence interval and p-value for both undiagnosed hypertension and uncontrolled hypertension while adjusting for individual clinicodemographic characteristics. Adjusting for Race, Ethnicity, sex, age, health insurance, valid range BMI, and CCI

## Results

### Characteristics of patients with undiagnosed hypertension

Out of 24,915 primary care patients with hypertension, 28.6% (*n* = 7,124) had undiagnosed hypertension, with a mean (SD) age of 49.3 (16.3) years (Table 1). Majority of patients within this cohort were 51.6% (*n* = 2,911) aged 18–44 years old, 28.7% (*n* = 3,458) males, 31.4% (*n* = 132) identified as Native Hawaiian/Pacific Islander race, 27.8% (*n* = 186) identified as Black/African American race, 32.6% (*n* = 1,007) identified as Hispanic/Latino ethnicity, 40.6% (*n* = 1,348) healthy weight patients, and 45.0% (*n* = 3,439) with a CCI of 0.

For other metabolic conditions studied, 7.6% (*n* = 508) of patients with dyslipidemia, 5.3% (*n* = 18) with LDL-c ≥ 190 mg/dl, 22.4% (*n* = 267) with LDL-c 70–189 mg/dl, and 9.2% (*n* = 588) of patients with hypercholesterolemia had undiagnosed hypertension.

### Risk factors of undiagnosed hypertension

After adjustments, significantly higher odds of undiagnosed hypertension were observed among younger patients 18–44 years (2.05 [1.90–2.21]), Hispanic/Latino ethnicity (1.13 [1.03–1.23]), covered by UT Medicaid (1.43 [1.29–1.58]), self-paid insurance status (1.32 [1.13–1.53]), CCI of 1–2 (1.79 [1.67–1.92]), and patients with LDL-c ≥ 190 mg/dl (3.05 [1.41–6.59]) (Table 2; Fig. 2).

On the other hand, significantly lower odds of undiagnosed hypertension were identified among patients aged 65 years and older (0.64 [0.57–0.71]), males (0.93 [0.88–0.99]), self-identified Asian race (0.65 [0.53–0.80]) or Black/African American race (0.61 [0.50–0.74]), overweight BMI (0.81 [0.74-0.89]), obesity in class I (0.75 [0.71-0.79]), class II (0.76 [0.68-0.85]), or class III (0.67 [0.60-0.75]), CCI of 3-4 (0.57 [0.51-0.63]) or CCI of 5–21 (0.36 [0.32–0.41]), statin users (0.72 [0.67–0.77]), with diabetes (0.37 [0.33–0.40]), controlled HbA1c (0.42 [0.37–0.47]), with dyslipidemia (0.25 [0.22–0.27]), LDL-c 70–189 mg/dl (0.74 [0.63–0.86]), and hypercholesterolemia (0.28 [0.25–0.31]) (Table 2; Fig. 2).

### Black/African American vs. White patients

In all cases of statistically significant results, the odds of patients with hypertension having undiagnosed hypertension were lower among Black/African Americans than their White counterparts (Table 3). The lowest odds of undiagnosed hypertension in Black/African Americans exist for geriatric age 65+ (0.43 [0.21–0.79]), female (0.61 [0.46–0.79]),overweight BMI (0.78 [0.38-0.93]), CCI of 3–4 (0.38 [0.17–0.73]), controlled HbA1c levels (0.17 [0.04–0.47]), and hypercholesterolemia (0.18 [0.03–0.57]) (Table 3; Fig. 3).

**Table 3:** Risk factors of undiagnosed hypertension – Black/African American (*N* = 668) vs. White (*n* = 19,671)

Risk Factors	Undiagnosed Hypertension				Difference %	Adjusted OR [95% CI]	*P* Values^1^
	Black/African American		White				
	**N**	Yes (%)	**N**	Yes (%)			
Age (years) mean (SD)	51.3 (15.0)	41.5 (13.4)	58.3 (16.3)	49.6 (16.5)		**-**	**-**
Age							
18–44 years	226	109 (48.2)	4,296	2,224 (51.8)	-4	**0.72 [0.54–0.95]**	**0.022**
45–64 years	305	67 (21.9)	7,509	2,087 (27.8)	-6	**0.66 [0.49–0.88]**	**0.005**
65 + years	137	10 (7.2)	7,866	1,174 (14.9)	-8	**0.43 [0.21–0.79]**	**0.013**
Sex							
Female	333	94 (28.2)	10,076	2,830 (28.1)	0	**0.61 [0.46–0.79]**	**< 0.001**
Male	335	92 (27.4)	9,592	2,653 (27.7)	-1	**0.71 [0.54–0.92]**	**0.012**
BMI (kg/m^2^) mean (SD)	31.7 (7.9)	31.3 (7.9)	31.3 (7.8)	30.8 (7.9)		-	-
BMI (kg/m^2^)							
Underweight (< 18.5)	3	1	167	61 (36.5)	-	**-**	**-**
Healthy Weight (18.5–24.99)	114	41 (35.9)	3,221	1,181 (36.6)	-1	**0.66 [0.41-2.89]**	**0.551**
Overweight (25.0–29.99)	141	38 (26.9)	4,624	1,359 (29.4)	-2	**0.78 [0.38-0.93]**	**0.002**
Obesity Class 1 (30.0–34.99)	165	46 (27.9)	4,278	1,166 (27.3)	1	**0.82 [0.74-0.97]**	**0.019**
Obesity Class 2 (35.0–39.99)	89	22 (24.7)	2,324	643 (27.7)	-3	0.91 [0.52–1.56]	0.737
Obesity Class 3 (40.0+)	74	22 (29.7)	2,119	538 (25.4)	5	1.08 [0.58–1.88]	0.808
Insurance							
UT Commercial	323	106 (32.8)	9,550	3,270 (34.2)	-1	0.86 [0.67–1.11]	0.246
UT Medicare	141	13 (9.2)	7,905	1,251 (15.8)	-7	**0.45 [0.23–0.79]**	**0.009**
UT Medicaid	152	45 (29.6)	1,501	687 (45.8)	-16	**0.38 [0.25–0.56]**	0.009
Self-pay	43	17 (39.5)	562	215 (38.3)	2	0.91 [0.45–1.78]	0.779
CCI							
None (0)	221	107 (48.4)	5,978	2,629 (44.0)	4	0.87 [0.66–1.16]	0.353
Mild (1–2)	249	58 (23.2)	6,866	1,966 (28.6)	-6	**0.51 [0.37–0.70]**	**< 0.001**
Moderate (3–4)	98	9 (9.1)	3,311	550 (16.6)	-8	**0.38 [0.17–0.73]**	**0.007**
Severe (5–21)	97	11 (11.3)	3,497	333 (9.5)	2	0.96 [0.47–1.79]	0.901
Current Statin Use							
Yes	229	33 (14.4)	7,789	1,216 (16.2)	-2	**0.80 [0.58-1.07]**	**0.136**
Diabetes							
Diabetes ICD (Yes)	197	13 (6.5)	4,736	444 (9.4)	-2	**0.41 [0.20–0.75]**	**0.007**
Pre-Diabetes (Yes)	95	25 (26.3)	2,127	522 (24.5)	2	0.75 [0.45–1.24]	0.277
Blood Glucose Levels							
Controlled HbA1c ICD (Yes)	115	4 (3.4)	3,220	287 (8.9)	-6	**0.17 [0.04–0.47]**	**0.003**
Other Metabolic Conditions							
Dyslipidemia (Yes)	122	-	5,328	404 (7.6)	-	-	-
LDLC_HIGH (> 190 mg/dl) (Yes)	0	-	27	14 (51.8)	-	-	-
LDLC_70_189 mg/dl (Yes)	23	4 (17.4)	932	194 (20.8)	-4	1.05 [0.27–3.31]	0.942
Hypercholesterolemia (Yes)	113	3 (2.6)	5,159	455 (8.8)	-6	**0.18 [0.03–0.57]**	**0.017**

Abbreviations: aOR, adjusted odds ratio; SD: Standard Deviation; BMI: Body mass index; CCI: Charlson comorbidity index; ICD: International classification of diseases (ICD); HbA1c: Hemoglobin A1c tests; LDL-c: Low-density lipoprotein; ^1^ Adjusted for age, sex, BMI, CCI and health insurance

### Characteristics of patients with uncontrolled hypertension

Of the 24,915 patients with hypertension, 37.4% (*n* = 9,319) had uncontrolled hypertension, with mean age of 57.9 (16.2) years (Table 1). Patients with uncontrolled hypertension were predominantly middle age 45–64 years (37.8%; *n* = 3,690), male (40%; *n* = 4,814), Native Hawaiian/Pacific Islander race (44.9%; *n* = 189), unknown BMI status (100%; *n* = 4,339), and patients with a CCI of 0 (43.2%; *n* = 3,304).

Regarding other medical conditions studied, 34.6% (*n* = 2,298) of patients with dyslipidemia, 37.8% (*n* = 451) with LDL-c 70–189 mg/dl, 35.3% (*n* = 12) with LDL-c ≥ 190 mg/dl, and 34.7% (*n* = 2,219) with hypercholesterolemia also had uncontrolled hypertension (Table 1).

### Risk factors of uncontrolled hypertension

Factors significantly associated with increased odds of uncontrolled hypertension included geriatric age 65 and older (1.11 [1.08–1.34]), male (1.24 [1.17–1.31]), self-identified Native Hawaiian/Pacific Islander race (1.32 [1.05-1.62]) or Black/African American race (1.24 [1.11-1.57]), and self-pay insurance (1.11 [1.03-1.22]). 

In contrast, patients with a CCI of 3–4 (0.70 [0.63-0.77]), current statin users (0.76 [0.72–0.81]), diabetic (0.86 [0.80–0.92]), or pre-diabetic (0.89 [0.82–0.97]), controlled HbA1c (0.84 [0.78–0.91]), dyslipidemia (0.85 [0.79–0.91]), and hypercholesterolemia (0.90 [0.89–0.90]), all exhibited significantly lowest odds of uncontrolled hypertension (Table 2; Fig. 4).

### Black/African American vs. White patients

For all significant results, the odds of uncontrolled hypertension were higher for Black/African American patients than for White patients (Table 4; Fig. 5). The odds of uncontrolled hypertension were highest in younger Black/African Americans 18–44 years (1.42 [1.10–1.90]), female (1.41 [1.12–1.72]), obesity in class I (1.43 [1.19–1.62]), medicaid insurance (1.48 [1.03-2.11]), CCI of 3–4 (1.97 [1.29–2.98]), diabetic (1.51 [1.08–2.09]), statin user (1.41 [1.04–1.89]), and controlled HbA1c (1.74 [1.14–2.61]).

**Table 4:** Risk factors of uncontrolled hypertension – Black/African American (*N* = 668) vs. White (*n* = 19,671)

Risk Factors	Uncontrolled Hypertension				Difference %	Adjusted OR [95% CI]	*P* Values^1^
	Black/African American		White				
	**N**	Yes (%)	**N**	Yes (%)			
Age (years) mean (SD)	51.3 (15.0)	51.3 (14.1)	58.3 (16.3)	58.7 (16.3)		**-**	**-**
Age							
18–44 years	226	92 (40.7)	4,296	1,553 (36.1)	4.6	**1.42 [1.10–1.90]**	**0.007**
45–64 years	305	133 (43.6)	7,509	2,808 (37.4)	6.2	**1.25 [1.12–1.34]**	**0.011**
65 + years	137	59 (43.1)	7,866	2,859 (36.3)	6.7	1.19 [0.82–1.71]	0.342
Sex^2^							
Female	333	137 (41.1)	10,076	3,472 (34.5)	6.7	**1.41 [1.12–1.72]**	**0.002**
Male	335	147 (43.9)	9,592	3,747 (39.1)	4.8	**1.21 [1.03–1.55]**	**0.044**
BMI (kg/m^2^) mean (SD)^3^	31.7 (7.9)	32.0 (8.2)	31.3 (7.8)	31.4 (7.7)			
BMI (kg/m^2^)							
Underweight (< 18.5)	3	1	167	52 (31.1)	2.2	-	-
Healthy Weight (18.5–24.99)	114	43 (37.7)	3,221	1,008 (31.3)	6.4	1.45 [0.97–2.15]	0.664
Overweight (25.0–29.99)	141	56 (39.7)	4,624	1,502 (32.5)	7.2	**1.21 [1.04-1.41]**	**0.012**
Obesity Class 1 (30.0–34.99)	165	66 (40.0)	4,278	1,432 (33.5)	6.5	**1.43 [1.19–1.62]**	**<0.001**
Obesity Class 2 (35.0–39.99)	89	35 (39.3)	2,324	736 (31.7)	7.7	1.44 [0.92–2.23]	0.102
Obesity Class 3 (40.0+)	74	29 (39.2)	2,119	666 (31.4)	7.8	1.33 [0.80–2.15]	0.257
Insurance^3^							
UT Commercial	323	141 (43.7)	9,550	3,639 (38.1)	5.5	**1.30 [1.11–1.71]**	**0.009**
UT Medicare	141	58 (41.1)	7,905	2,765 (35.0)	6.2	1.28 [0.89–1.82]	0.172
UT Medicaid	152	62 (40.8)	1,501	502 (33.4)	7.3	**1.48 [1.03–2.11]**	**0.031**
Self-pay	43	22 (51.2)	562	263 (46.8)	4.4	1.32 [0.70–2.70]	0.310
CCI^4^							
None (0)	221	103 (46.6)	5,978	2,505 (41.9)	4.7	**1.41 [1.06–1.86]**	**0.016**
Mild (1–2)	249	101 (40.6)	6,866	2,497 (36.4)	4.2	1.19 [0.95–1.30]	0.110
Moderate (3–4)	98	47 (48.0)	3,311	1,101 (33.3)	14.7	**1.97 [1.29–2.98]**	**0.001**
Severe (5–21)	97	31 (32.0)	3,497	1,104 (31.6)	0.4	1.18 [0.75–1.82]	0.458
Current Statin Use							
Yes	229	93 (40.6)	7,789	2,626 (33.7)	6.9	**1.41 [1.04–1.89]**	**0.025**
Diabetes							
Diabetes ICD (Yes)	197	81 (41.1)	4,736	1,526 (32.2)	8.9	**1.51 [1.08–2.09]**	**0.013**
Pre-Diabetes (Yes)	95	38 (40.0)	2,127	687 (32.3)	7.7	1.31 [0.95-1.42]	0.121
Blood Glucose Levels							
Controlled HbA1c ICD (Yes)	115	46 (40.0)	3,220	944 (29.3)	10.7	**1.74 [1.14–2.61]**	**0.009**
Other Metabolic Conditions							
Dyslipidemia (Yes)	122	45 (36.9)	5,328	1,815 (34.1)	2.8	1.22 [0.81–1.81]	0.340
LDLC_HIGH (> 190 mg/dl) (Yes)	0	0	27	9 (33.3)	-	-	-
LDLC_70_189 mg/dl (Yes)	23	9 (39.1)	932	360 (38.6)	0.5	1.14 [0.41-3.00]	0.794
Hypercholesterolemia (Yes)	113	39 (34.5)	5,159	1,749 (33.9)	0.6	1.13 [0.72–1.72]	0.585

Abbreviations: aOR, adjusted odds ratio; SD: Standard Deviation; BMI: Body mass index; CCI: Charlson comorbidity index; ICD: International classification of diseases (ICD); HbA1c: Hemoglobin A1c tests; LDL-c: Low-density lipoprotein; ^1^ Adjusted for age, sex, BMI, CCI and health insurance as applicable; ^2^ There were an additional 3 cases of reported sex “other”; ^3^ For 193 patients “Other Insurance” has been documented. ^4^ CCI unknown for 31 patients

## Discussion

This study aimed to identify factors associated with undiagnosed and uncontrolled hypertension in primary care patients with hypertension. The study also examined whether Black/African Americans are at higher odds of undiagnosed and uncontrolled hypertension compared to White patients.

There are several key findings. First, younger patients, Hispanic/Latino ethnicity, a mild (1–2) comorbidity score, and patients with LDL-c ≥ 190 mg/dl had significantly higher odds of undiagnosed hypertension. Second, geriatric age (65+), self-identified Black/African American race, self-identified Native Hawaiian/Pacific Islander race, and mild (1–2) comorbidity score was associated with significantly higher odds of uncontrolled hypertension.

Finally, Black/African Americans had much lower odds of undiagnosed hypertension and higher odds of uncontrolled hypertension than White patients. In Black/African Americans, the lowest odds of undiagnosed hypertension occurred when they were older, female, had a overweight weight BMI, moderate (3–4) CCI score, had controlled HbA1c, and were diagnosed with hypercholesterolemia. For uncontrolled hypertension, Black/African Americans had the highest odds when they were younger, female, class I obese, medicaid insurance, moderate (3–4) CCI score, currently using statins, diabetic, and had controlled HbA1c.

More in detail, in this study, younger age was associated with increased odds of undiagnosed hypertension. Prior studies support this finding [21–23]. Poor adherence to clinic visits contributes to this problem [22] and implies limited opportunities for physicians to perform diagnostic procedures, such as BP readings. There may also be limited BP screenings due to younger populations generally perceived as having better health compared to other age groups [21]. Limited BP screenings have also been linked to provider concerns about giving false hypertension diagnoses due to increased BP variability in this age group [22].

The present study also identified higher odds of undiagnosed hypertension among patients of Hispanic/Latino ethnicity. A particularly concerning finding is the scarcity of research on the risks and consequences of undiagnosed hypertension within the Hispanic/Latino community, especially in primary care. Nevertheless, current related research indicates significantly lower odds of hypertension awareness, treatment, and control in Hispanics/Latino individuals [8]. The persistent effects of health illiteracy, insufficient insurance coverage, and limited culturally and linguistically competent healthcare workforce have been documented [8, 24, 25].

Another important finding is the increased odds of undiagnosed hypertension among patients with low comorbidity scores. Higashi et al. (2007) [26] reported that the quality of care improved as the number of medical conditions increased. For the authors, this association is strengthened by the higher healthcare utilization rates in patients with multiple health conditions and the increased involvement of multiple healthcare specialists in their care. As a result, there is a higher likelihood of early diagnosis of other health conditions. These results reveal that the presence of multiple comorbidities should not be the sole basis for hypertension screenings, as this may lead to disparities in identifying at risk patients.

The present study also found that patients with LDL-c ≥ 190 mg/dl had significantly higher odds of undiagnosed hypertension. This finding is surprising given that hypertension is a potential outcome resulting from very high LDL-c levels [27]. A potential explanation is the phenomenon of treating the most disruptive symptoms first [28]. In this case, it is possible that clinical interventions primarily sought to bring the high LDL-c level to control and considered other comorbidities or outcomes (e.g. hypertension) to be a secondary intervention focus.

Lastly, patients covered by Medicaid insurance and those who are self-paying both had increased risk of undiagnosed and uncontrolled hypertension. Importantly, these populations are likely to be low-income. A possible explanation for the observed odds is that due to affordability concerns, low-income and uninsured patients may choose not to seek care, return for follow-up visits, or complete treatment programs, resulting in limited screening and hypertension confirmation opportunities, and ultimately poor hypertension control [21]. Inadequate income and insurance may also impact eligibility for clinical care programs, which can impact physician’s ability to provide preventive care and evidence-based clinical interventions or prescribe medications that may only be covered by comprehensive insurance [21, 29].

Concerning the second study aim, this study showed a significantly lower likelihood of undiagnosed hypertension in Black/African American patients than in white patients, regardless of which risk factors were considered. This result is not entirely surprising, as greater hypertension awareness is being documented in Black/African Americans compared to White individuals [8, 28]. Extensive hypertension outreach and screening opportunities due to increased vigilance within healthcare systems on the prevalence of hypertension in Black/African Americans help explain this trend [28].

Unfortunately, lower undiagnosed hypertension rates in Black/African Africans does not translate to improved hypertension control and management. In the present study, there were significantly higher risk of uncontrolled hypertension in Black/African Americans and Native Hawaiian/Pacific Islanders compared to White primary care patients. Comparative studies of uncontrolled hypertension in Black/African Americans within US primary care settings are very limited, and many previous studies have focused on the overall population [6, 8, 30–33]. These studies point to poor adherence to hypertension medications and treatments [30, 31]. insurance instability [33], psychosocial stressors [32], and resistant hypertension [33], as major reasons for poor hypertension control and management.

For Native Hawaiian/Pacific Islander race, poor healthcare and sedentary lifestyles are significant risk factors for uncontrolled hypertension [34]. A major concern here is that Native Hawaiian/Pacific Islander individuals are severely underrepresented in primary care research and are highly affected by inadequate access to primary care services [35].

Furthermore, the present study revealed significantly higher odds of uncontrolled hypertension in older primary care patients. This finding has been documented in prior studies [36, 37]. Certain system changes occurring in older age are a potential pathway for uncontrolled hypertension. These changes include decreased cardiovascular capacities due to stiffening of the arterial walls, which causes difficulties in maintaining BP [36]. Another pathway is the lack of intensified hypertension treatment. Although research generally points to better BP outcomes from treatment intensification in geriatric age [38, 39], the risk of over-treatment, undue patient burden and lack of standardized guidelines have hindered adoption [40].

Finally, the results showing increased odds of uncontrolled hypertension in males compared to females are also reported in some studies [41–44]. Some contributing factors are poor medication adherence and lower healthcare utilization [43, 44]. Other related studies have reported higher odds in females [45, 46]. The conflicting findings may be due to the moderating role of aging and race. A 2017 American Heart Association (AHA) report revealed a higher prevalence of hypertension in women after age 64, and older Black/African American women having even higher rates [47]. The report considered medication access and health disparities as influential factors.

In summary, primary care can be essential in addressing hypertension disparities. A major defining feature is its community and patient-centered model of care, which prioritizes affordability and prevention. This model of care can help increase the reach and capacity of health services, especially in underserved communities. However, more research is needed on the best practices for accurately identifying patients with hypertension and developing culturally tailored hypertension management programs for those patients.

### Strengths & limitations

This study is the first in Utah to provide an in-depth overview of the risk factors associated with undiagnosed and uncontrolled hypertension in primary care. It also fills a research gap by drawing attention to hypertension disparities in primary care. In this way, the study informs clinical approaches to better identify and address undiagnosed and uncontrolled hypertension in primary care.

The study also has limitations. The study is based on an academic primary care patient population sample. The data may not be representative of the general population, such as those receiving care from non-academic and publicly funded health systems. Additionally, the study did not include data on educational level. Existing research has found a higher incidence of hypertension among individuals with lower education attainment [32]. Therefore, the results of the present study may not be generalizable to those individuals. Further, the study analysis did not account for the 2020 AHA guidelines on lower BP assessment in older adults with diabetes [48], which may overestimate the odds of uncontrolled hypertension given comorbidity of diabetes. In addition, antihypertensive medication is also used to treat other conditions, which may have led to overestimating the odds of uncontrolled hypertension. Finally, the study design is cross-sectional. This study design makes it difficult to establish a cause-and-effect relationship, since analysis is done at one point in time.

## Conclusions

The results of this study suggest that undiagnosed and uncontrolled hypertension is prevalent in primary care and that disparities exist. Critical risk factors for undiagnosed hypertension include younger age, Hispanic/Latino ethnicity, very high LDL-c, and low comorbidity scores. For uncontrolled hypertension, geriatric populations, males, Native Hawaiian/Pacific Islanders, and Black/African Americans, continue to experience greater burdens than their counterparts. Substantial efforts are needed to strengthen hypertension diagnosis and to develop tailored hypertension management programs in primary care, focusing on these populations.

### Electronic supplementary material

Below is the link to the electronic supplementary material.

Supplementary Material 1

Supplementary Material 2

## Data Availability

Study data is owned by the University of Utah (UofU), Salt Lake City Utah. Data requests should be made to Dominik Ose, the corresponding author. The corresponding author will then forward the request to the UofU software licensing office who will process the request, including issuing a data use agreement with the requesting party and providing relevant access information.
