# Supplementary material for: Risk factors of undiagnosed and uncontrolled hypertension in primary care patients with hypertension: a cross-sectional study
Source: BMC Prim Care. 2024 Aug 20;25:311. doi: 10.1186/s12875-024-02511-4 (PMC11334361; doi:10.1186/s12875-024-02511-4)
Supplement: Supplementary file 1 — Supplementary Material 1 [file 12875_2024_2511_MOESM1_ESM.docx]

**Supplementary Tables**

**eTable 1: Risk Factors of Undiagnosed and Uncontrolled Hypertension in Primary Care Patients; Crude Odds ratio**

**eTable 2: Risk Factors of Undiagnosed and Uncontrolled Hypertension– Black/African American (N=668) vs. White (n=19,671); Crude Odds Ratio**

**eTable 1: Risk Factors of Undiagnosed and Uncontrolled Hypertension in Primary Care Patients; Crude Odds ratio**

|  | Undiagnosed Hypertension | | | Uncontrolled Hypertension | | |
| --- | --- | --- | --- | --- | --- | --- |
| Risk Factors | **OR** | **95% CI** | ***P-*values** | **OR** | **95% CI** | ***P-*values** |
| Age |  |  |  |  |  |  |
| 45-64 years old | Ref=1 | | | | | |
| 18-44 years old | 2.70 | [2.52-2.89] | <0.001 | 1.02 | [0.94-1.12] | 0.548 |
| 65+ years old | 0.46 | [0.42-0.49] | <0.001 | 1.03 | [0.96-1.11] | 0.299 |
| Sex |  |  |  |  |  |  |
| Female | Ref=1 | | | | | |
| Male | 1.01 | [0.96-1.07] | 0.684 | 1.22 | [1.17-1.39] | <0.001 |
| Race |  |  |  |  |  |  |
| White | Ref=1 | | | | | |
| Asian | 0.93 | [0.79-1.10] | 0.420 | 1.09 | [0.98-1.21] | 0.056 |
| Black/African American | 0.98 | [0.83-1.17] | 0.857 | 1.35 | [1.14-1.58] | <0.001 |
| Native-Hawaiian/Other Pacific Islander | 1.17 | [0.94-1.43] | 0.150 | 1.37 | [1.11-1.67] | 0.002 |
| American Indian/Alaska Native | 1.06 | [0.77-1.47] | 0.704 | 0.83 | [0.59-1.15] | 0.274 |
| Other | 1.12 | [1.03-1.22] | 0.007 | 1.17 | [1.07-1.27] | <0.001 |
| Ethnicity |  |  |  |  |  |  |
| Non-Hispanic/Latino | Ref=1 | | | | | |
| Hispanic/Latino | 1.25 | [1.15-1.35] | <0.001 | 1.04 | [0.95-1.16] | 0.231 |
| BMI kg/m^2^ |  |  |  |  |  |  |
| Healthy weight (18.5-24.99) | Ref=1 | | | | | |
| Underweight (<18.5) | 1.04 | [0.77-1.40] | 0.795 | 1.06 | [0.80-1.45] | 0.697 |
| Overweight (25.0 – 29.99) | 0.77 | [0.70-0.84] | <0.001 | 1.04 | [0.96-1.11] | 0.339 |
| Obesity Class 1 (30.0-34.99) | 0.70 | [0.64-0.76] | <0.001 | 1.09 | [1.00-1.19] | 0.053 |
| Obesity Class 2 (35.0-39.99) | 0.69 | [0.63-0.77] | <0.001 | 1.05 | [0.95-1.16] | 0.353 |
| Obesity Class 3 (40+) | 0.64 | [0.57-0.71] | <0.001 | 1.03 | [0.93-1.15] | 0.572 |
| Insurance |  |  |  |  |  |  |
| UT Commercial | Ref=1 | | | | | |
| UT Medicare | 0.35 | [0.33-0.37] | <0.001 | 0.92 | [0.90-0.97] | 0.031 |
| UT Medicaid | 1.37 | [1.25-1.50] | <0.001 | 0.90 | [0.83-0.96] | 0.001 |
| Self-Pay | 1.35 | [1.17-1.54] | <0.001 | 1.11 | [1.08-1.18] | <0.001 |
| CCI |  |  |  |  |  |  |
| Mild | Ref=1 | | | | | |
| None | 1.99 | [1.86-2.12] | <0.001 | 1.17 | [1.09-1.25] | <0.001 |
| Moderate | 0.48 | [0.44-0.53] | <0.001 | 0.64 | [0.53-0.77] | <0.001 |
| Severe | 0.27 | [0.24-0.30] | <0.001 | 0.68 | [0.55-0.85] | <0.001 |
| Statins |  |  |  |  |  |  |
| Statin 2020 (yes) | 0.37 | [0.35-0.40] | <0.001 | 0.81 | [0.76-0.86] | <0.001 |
| Diabetes |  |  |  |  |  |  |
| Diabetes_ICD (yes) | 0.21 | [0.19-0.23] | <0.001 | 0.82 | [0.77-0.87] | <0.001 |
| Pre-Diabetes (yes) | 0.90 | [0.82-0.99] | 0.022 | 0.89 | [0.85-0.96] | <0.001 |
| Blood Glucose Level |  |  |  |  |  |  |
| HbA1c_controlled_ICD (yes) | 0.22 | [0.20-0.24] | <0.001 | 0.83 | [0.75-0.89] | <0.001 |
| Other Metabolic Conditions |  |  |  |  |  |  |
| Dyslipidemia (yes) | 0.15 | [0.13-0.16] | <0.001 | 0.85 | [0.80-0.90] | <0.001 |
| LDLC_level_vHIGH (yes) | 2.81 | [1.42-5.60] | 0.002 | 1.12 | [0.52-2.27] | 0.765 |
| LDLC_level_70_189 (yes) | 0.71 | [0.62-0.81] | <0.001 | 1.11 | [0.98-1.26] | 0.096 |
| Hypercholesterolemia (yes) | 0.19 | [0.17-0.20] | <0.001 | 0.86 | [0.80-0.91] | <0.001 |

Crude Odds ratio

**eTable 2: Risk Factors of Undiagnosed and Uncontrolled Hypertension– Black/African American (N=668) vs. White (n=19,671); Crude Odds Ratio**

|  | Undiagnosed Hypertension | | | Uncontrolled Hypertension | | |
| --- | --- | --- | --- | --- | --- | --- |
| Risk Factors | **OR** | **95% CI** | ***P-*values** | **OR** | **95% CI** | ***P-*values** |
| Age |  |  |  |  |  |  |
| 45-64 years old | **Ref** | | | | | |
| 18-44 years old | 1.39 | [1.05-1.83] | 0.020 | 1.40 | [1.10-1.78] | 0.006 |
| 65+ years old | 2.26 | [1.18-4.31] | 0.014 | 1.20 | [0.84-1.72] | 0.319 |
| Sex |  |  |  |  |  |  |
| Female | **Ref** | | | | | |
| Male | 0.95 | [0.59-1.52] | 0.819 | 1.74 | [1.12-2.70] | 0.014 |
| Ethnicity |  |  |  |  |  |  |
| Non-Hispanic/Latino | **Ref** | | | | | |
| Hispanic/Latino | 1.26 | [0.51-3.11] | 0.622 | 2.57 | [1.06-6.27] | 0.037 |
| BMI kg/m^2^ |  |  |  |  |  |  |
| Healthy weight (18.5-24.99) | **Ref** | | | | | |
| Underweight (<18.5) | 0.87 | [0.08-9.78] | 0.909 | 1.09 | [0.22-4.47] | 0.899 |
| Overweight (25.0 – 29.99) | 0.87 | [0.60-1.27] | 0.483 | 1.30 | [1.07-1.61] | 0.007 |
| Obesity Class 1 (30.0-34.99) | 1.02 | [0.72-1.44] | 0.913 | 1.20 | [1.05-1.26] | 0.022 |
| Obesity Class 2 (35.0-39.99) | 0.84 | [0.67-1.06] | 0.152 | 1.03 | [0.92-1.16] | 0.600 |
| Obesity Class 3 (40+) | 0.84 | [0.66-1.06] | 0.149 | 1.01 | [0.90-1.14] | 0.852 |
| Insurance |  |  |  |  |  |  |
| UT Commercial | **Ref** | | | | | |
| UT Medicare | 0.53 | [0.30-0.94] | 0.031 | 1.23 | [0.86-1.75] | 0.254 |
| UT Medicaid | 2.04 | [1.42-2.94] | <0.001 | 1.50 | [1.06-2.13] | 0.022 |
| Self-Pay | 0.96 | [0.51-1.81] | 0.905 | 1.05 | [0.86-1.52] | 0.622 |
| CCI |  |  |  |  |  |  |
| Mild | **Ref** | | | | | |
| None | 1.18 | [0.90-1.54] | 0.236 | 1.42 | [1.08-1.87] | 0.012 |
| Moderate | 0.50 | [0.25-1.01] | 0.052 | 1.84 | [1.22-2.78] | 0.004 |
| Severe | 1.20 | [0.63-2.26] | 0.583 | 1.13 | [0.73-1.75] | 0.582 |
| Statins |  |  |  |  |  |  |
| Statin 2020 (yes) | 0.86 | [0.59-1.25] | 0.427 | 1.52 | [1.15-2.00] | **0.003** |
| Diabetes |  |  |  |  |  |  |
| Diabetes_ICD (yes) | 0.67 | [0.38-1.19] | 0.171 | 1.46 | [1.08-1.98] | 0.013 |
| Pre-Diabetes (yes) | 1.08 | [0.68-1.72] | 0.318 | 1.42 | [0.92-2.18] | 0.115 |
| Blood Glucose Level |  |  |  |  |  |  |
| HbA1c_controlled_ICD (yes) | 0.36 | [0.13-0.99] | 0.048 | 1.73 | [1.18-2.54] | 0.005 |
| Other Metabolic Conditions |  |  |  |  |  |  |
| Dyslipidemia (yes) | 0.10 | [0.01-0.71] | 0.021 | 1.31 | [0.89-1.92] | 0.164 |
| LDLC_level_vHIGH (yes) | - | - | - | - | - | - |
| LDLC_level_70_189 (yes) | 0.78 | [0.26-2.32] | 0.656 | 1.06 | [0.44-2.53] | 0.900 |
| Hypercholesterolemia (yes) | 0.28 | [0.09-0.87] | 0.028 | 1.17 | [0.78-1.75] | 0.457 |
