# Supplementary material for: Risk factors of undiagnosed and uncontrolled hypertension in primary care patients with hypertension: a cross-sectional study
Source: BMC Prim Care. 2024 Aug 20;25:311. doi: 10.1186/s12875-024-02511-4 (PMC11334361; doi:10.1186/s12875-024-02511-4)
Supplement: Supplementary file 2 — Supplementary Material 2 [file 12875_2024_2511_MOESM2_ESM.docx]

**DATA DESCRIPTION**

**Patient population:**

All patients with at least on primary care office/Telemedicine visit (internal medicine, family medicine; peds excluded), in Primary Care between **01/01/2020** and **12/31/2020** and who are 18 years and older.

**Variables:**

**Patient and clinic related variables**

| **Name** | **Description** | **Example** | **Logic** |
| --- | --- | --- | --- |
| **Race** | Race of patient | Black/African American |  |
| **Ethnicity** | Ethnicity of patient | Hispanic/Latino |  |
| Lang_pref | Primary language spoken by patient | English, Spanish, Other |  |
| Health_insur | Primary health insurance | Medicare, Medicaid, Private/Commercial, Self-Pay/No Insurance | Insurance for the most recent primary care visit in 2020 |
| DOB | Date of birth | 08/09/1979 |  |
| Age | Patient age in years | 36 | Age at the most recent visit |
| Sex | Patient’s biological sex | Female, male |  |
| Patient_county | Patient county of residence | Salt Lake County |  |
| Patients_zip | Patient zip code | 84103 | Zipcode for the most recent primary care visit in 2020 |
| Clinic_name | Name of facility that patient receives primary care services. | Madsen Clinic | Clinic for the most recent PCP visit in 2020 |
| CCI_latest | Latest Charleston Comorbidity Index | 2 | From most recent PCP office visit in 2020 |
| PCP | Designated PCP or if no PCP is designated the last provider | Provider9 | Latest provider of those primary care visit if PCP is null |
| Obesity (categorized) | 1=”Class 1” (BMI of 30 to < 35); 2=”Class 2” (BMI of 35 to < 40); 3=”Class 3” (BMI of 40 or higher) | 3 | Latest available in 2020 |
| BMI (if not available use latest weight and height to calculate BMI) | Calculated out of the EHR | 31.1 | Most recent BMI (calculated by EPIC) available for any visit |

**Hypertension related variables**

| **Name** | **Description** | **Logic** |
| --- | --- | --- |
| Hypertension_ICD  (yes/no) | Definition of hypertension based on ICD Codes | 18 years of age or older, have had a hypertension diagnosis (ICD code) in the past 5 years in an encounter, in their problem list, or in an invoice or claim. |
| Hypertension_vitals  (yes/no) | Definition of hypertension based on blood pressure readings | 18 years of age or older and two or more blood pressure readings on different dates in 2020 where systolic is ≥140 or diastolic is ≥ 90 (not included emergency room visits) |
| Hypertension_med  (yes/no) | Definition of hypertension based on antihypertensive medication | 18 years of age or older and a prescription for an antihypertensive medication in 2020 |
| **Hypertension_OVERALL**  **(yes/no)** | Definition of hypertension based on ICD Codes, blood pressure readings and antihypertensive medication | Hypertension_ICD (yes) or Hypertension_vitals (yes) or Hypertension_med (yes) |
| **Hypertension_undiag**  **(yes/no)** | Definition of undiagnosed hypertension based on missing ICD Codes | Patients who have hypertension based on lab values (Hypertension_vitals=yes) or medication prescription (Hypertension_med=yes) but no ICD code (Hypertension_ICD=no). |
| Hypertension_uncon_ICD (yes/no) | Definition of uncontrolled hypertension based on ICD code and elevated blood pressure reading | Patient with hypertension (Hypertension_ICD) whose latest blood pressure reading in 2020 is systolic ≥140 or diastolic ≥ 90. |
| **Hypertension_uncon_overall (yes/no)** | Definition of uncontrolled hypertension based on hypertension overall and elevated blood pressure reading | Patient with hypertension (Hypertension_overall) whose latest blood pressure reading in 2020 is systolic ≥140 or diastolic ≥ 90. |
| Hypertension_con_ICD (yes/no) | Definition of controlled hypertension based on ICD code and normal blood pressure reading | Patient with hypertension (Hypertension_ICD) whose latest blood pressure reading in 2020 is systolic < 140 and diastolic < 90. |
| **Hypertension_con_overall (yes/no)** | Definition of controlled hypertension based on hypertension overall and normal blood pressure reading | Patient with hypertension (Hypertension_overall) whose latest blood pressure reading in 2020 is systolic < 140 and diastolic < 90. |
| ASCVD  (yes/no) | Diagnosis of ASCVD | Patients with an ICD code for “Atherosclerotic Cardiovascular Disease” *[ICD-10: I25.10]* |
| Rhabdomyolysis  (yes/no) | Diagnosis of Rhabdomyolysis | Patients with an ICD code for Rhabdomyolysis *[ICD-10: M62.82]* |
| End_stage_renal_disease  (yes/no) | Diagnosis of End Stage Renal Disease | Patients with an ICD code for end stage renal disease *[ICD-10: N18.5, N18.6, Z94, Z99.1] exclude them right from the start* |

**Statin related variables**

| **Name** | **Description** | **Logic** |
| --- | --- | --- |
| Dyslipidemia**  (yes/no) | Definition of Dyslipidemia | Patients with an ICD diagnosis for Dyslipidemia *[ICD-10:* E78.1, E78.2, E78.3, E78.4, E78.5, E78.6, E78.7, E78.8, E78.9*]* |
| LDLC_level_vHIGH**  (yes/no) | Definition of high LDL-C level | Patients who have ever had a fasting or direct laboratory result of  LDL-C >= 190 mg/dL |
| LDLC_level_70_189**  (yes/no) | LDL-C level between 70-189 mg/dL | Patients with a fasting or direct laboratory result for LDL-C between 70-189 mg/dL in 2018, 2019 or in 2020 |
| Hypercholesterolemia**  (yes/no) | Definition of hypercholesterolemia | Patients with an ICD diagnosis for Hypercholesterolemia *[ICD-10: E78.0, E78.00, E78.01, E78.1, E78.2, E78.6]* |
| Statin_2020**  (yes/no) | Definition of statin use in 2020 | Patients with a prescription of statin in 2020 *[Atorvastatin (Lipitor), Lovastatin (Altoprev), Pitavastatin (Livalo, Zypitamag), Pravastatin (Pravachol), Rosuvastatin (Crestor, Ezallor), Simvastatin (Zocor)]* |

**Diabetes related variables**

| **Name** | **Description** | **Logic** |
| --- | --- | --- |
| Diabetes  (yes/no) | Definition of diabetes | 18 years of age or older, have had a diabetes diagnosis (ICD code) in the past 5 years in an encounter, in their problem list, or in an invoice or claim. |
| Pre-Diabetes  (yes/no) | Definition of pre-diabetes | Patient with an ICD diagnosis (ICD-10: R73.xx) OR two or more elevated lab values on different dates in 2019 *[(HbA1c (%) 5.7-6.4 or Fasting plasma glucose (mg/dl) 100-125 or Blood glucose (mg/dl) 140-199)]* AND not met the indication for diabetes in 2020 or before. |
| HbA1c_controlled  (yes/no) | Definition of controlled HbA1c | Patient with a diabetes diagnosis (Diabetes_ICD) AND an HbA1c test *[HbA1c (%) ≤ 8.0]* in 2020. |
